# Family Medicine Residents' Confidence in Performing a Basic Obstetric Ultrasound at Primary Health Care Centres in Qassim Province, Saudi Arabia

**DOI:** 10.7759/cureus.76066

**Published:** 2024-12-20

**Authors:** Shoug Albadi, Chandra Sekhar

**Affiliations:** 1 Family Medicine, Family Medicine Academy, Qassim Health Cluster, Buraidah, SAU

**Keywords:** barriers, confidence, family medicine residents, fm residents, qassim, saudi arabia, suggestions, ultrasound

## Abstract

Background

The family medicine (FM) specialty is a link between the community and the hospital. FM residents performing ultrasounds, detecting problems early, and suggesting appropriate health intervention will reduce time and improve maternal health care as per the Saudi Commission for Health Specialties (SCFHS) and Saudi Vision 2030 initiative. The study's objectives are to find out the confidence of FM residents about basic ultrasound performance and the barriers associated with obstetric ultrasound at primary health care centers (PHCCs).

Methods

A cross-sectional study was conducted utilizing a self-administered questionnaire distributed via Google Forms from June 2024 to October 2024. A total of 62 FM residents were invited to participate, resulting in a response rate of 80.64% (n = 50). Data analysis was performed using SPSS v. 21.0 software (IBM Corp., Armonk, NY, US), where descriptive statistics were computed for continuous variables. Additionally, the chi-square test was employed to examine the relationship between residents' confidence in their psychomotor skills and the categorical variables of gender and residency level.

Results

In the current study, about 56% (n=28) received obstetric ultrasound (OBUS) training. Roughly 68% (n=34) were neutral about their confidence regarding the psychomotor skills associated with OBUS, and 6% (n=3) reported feeling confident in those skills. Nearly 24% (n=12) were cited as being uncertain about their confidence in OBUS's psychomotor skills; 9 residents were junior, and only 3 out of 12 were senior. Regarding the barriers encountered while performing OBUS, approximately 93.8% (n = 46) of residents reported a lack of training while 73% (n = 35) cited patient overload and insufficient trained instructors at the facility. In terms of suggestions from FM residents to improve their practice, 26.5% (n = 17) highlighted the necessity for additional obstetric ultrasound (OBUS) training, followed by 14% (n = 9) emphasizing the importance of having qualified doctors for OBUS instruction.

Conclusions

Based on the study results, FM residents require training, resource materials provision, specialist doctor training, and periodical retraining, as OBUS is a skill development procedure. According to the responses, senior residents tend to feel more assured in performing OBUS than their junior counterparts, likely due to their extensive practice and exposure.

## Introduction

The Kingdom of Saudi Arabia has more than 2,000 primary healthcare centers (PHCC) distributed around the country [1]. These centers play a major role in ensuring that access to quality health services, including antenatal care, is provided to all people in the local population. Hence, the Saudi Commission for Health Specialities directed the Saudi Board for Family Medicine (SBFM) programs to improve women's care at PHCCs, and a new initiative was taken to perform obstetric ultrasounds at the PHCCs. Prenatal care plays a vital role in better outcomes for the mother and the newborn, in identifying early complications.

Antenatal care is considered one of the most critical scopes of primary healthcare center’s services. The World Health Organization (WHO) guidelines on antenatal care recommend a minimum of eight contacts (active connection between a pregnant woman and a healthcare provider) to reduce perinatal mortality and improve women's care experiences. First contact takes place in the first trimester, two contacts in the second trimester, and five contacts in the third trimester. In addition, one ultrasound scan before 24 weeks of gestation (early ultrasound) is recommended for pregnant women to estimate gestational age, identify the location of pregnancy (e.g., intrauterine), confirm cardiac activity, known fetal size, assess chorionicity and amnionicity for multiple gestations, improve detection of fetal anomalies and multiple pregnancies, reduce the induction of labor for post-term pregnancy, and improve a woman's pregnancy experience. After 18 weeks of pregnancy, assessing fetal anatomy can be evaluated if the skill set and health systems allow: the presence of a normal head, neck, face, spine, chest, heart, abdomen, abdominal wall, extremities, placental appearance and location, and umbilical cord [2].

Throughout the globe, there is a shortage of obstetricians in rural and urban areas, including in developed countries [3]. The highest number of Saudi women are between 15 and 19 years of age, followed by the 20-24 years age group, as per the Saudi Women's Report 2022 [4]. When obstetric issues are identified at the PHCC, the physician builds confidence and establishes a comforting environment, unlike a hospital setting. Early intervention suggestions enhance the mother's outcomes.

There is a huge potential benefit to having an ultrasound performed by a family physician. It instantly identifies clinical problems and provides immediate care, indirectly reducing the time spent on the patient and healthcare costs. This will help achieve better outcomes in terms of reducing maternal and infant mortality rates in the community. It will indirectly help improve patient rapport and patient and physician satisfaction will increase tremendously [5].

Ultrasound is a safe and non-invasive method that allows instant access and optimum visualization of the problem [6]. Imaging ultrasound scans play a crucial role in obstetrics and are widely used for various purposes throughout pregnancy. Ultrasound imaging is highly beneficial for the proper diagnosis of life-threatening conditions that occur in the first trimester such as spontaneous abortion, gestational trophoblastic disorders (GTDs), and ectopic pregnancy. Y Wiafe et al. (2011) suggested that maternal mortality declined as a result of increased obstetric ultrasound usage and improved intervention [7].

The quality of the obstetric ultrasound examination is heavily operator-dependent. Therefore, ultrasound performance requires a component of knowledge, hands-on training, focused and repetitive practice, skills, and competency assessment. A recent systematic review by CA Andersen et al. (2019 showed that misinterpretations in ultrasonography may result in a wrong diagnosis, which could cause patients to be concerned and possibly delay intervention if an urgent condition is missed [8]. A study stated that family physicians' performance of ultrasound is widely included in FM residency programs and clarified the comparison between FM residents versus hospital radiologists for fetal biometry for gestational age determination in this study [9].

The American Academy of Family Physicians demonstrated that family medicine residents should be competent and comprehend in performing obstetric ultrasound in the following areas as part of maternity care: transabdominal and transvaginal scanning techniques, presence and viability of intrauterine pregnancy, detection of fetal heart rate, gestational age assessment, detection of free fluid in the pelvis, determine placenta position, fetal presentation, amniotic fluid volume, and identification of placenta abruption [10].

The Saudi Commission for Health Specialities (SCFHS) developed the Saudi Board for Family Medicine Curriculum 2022 stated that to enhance resident clinical skills and improve knowledge and confidence in essential family medicine domains, including antenatal ultrasound (fetal position, amniotic fluid index, placental location, cardiac activity). In addition, family residents should master the clinical knowledge required for the diagnosis of molar pregnancy, anencephalic pregnancy, placenta previa, fetal age, and ectopic pregnancy [11].

Based on the above circumstances, the study aims to assess confidence in performing basic obstetric ultrasounds among family residents in Qassim PHCCs and to determine the barriers to the performance of basic obstetric ultrasounds at Qassim PHCCs.

Objectives

The objectives are to assess the confidence among the FM residents about basic obstetric ultrasound performance in the study population, determine the barriers to the performance of the basic obstetric ultrasound and suggestions to improve OBUS at the PHCC, examine the factors associated with the ultrasound performance of FM residents in the study.

## Materials and methods

Study setting and target population

This study was conducted at the 13 primary healthcare centers in the Qassim region. The target population included family medicine residents working at the selected primary healthcare centers of Buraidah City and Unaizah City.

Study design

A cross-sectional study was conducted among Qassim family medicine residents through the online self-administered questionnaire.

Data collection

After constructing our research idea, we searched the literature and found about six studies. Then, when building the questionnaire, three studies were extensively studied [7,12,13], and the recommendations of the American Academy of Family Physicians (AAFP) and SCFHS were considered for the questionnaire's construction. After the questionnaire was constructed, it was validated by our institute's experienced research faculty and family medicine faculty.

Data collection was based on the questionnaire survey, which was emailed to Qassim family medicine residents (Appendices). The questionnaire was divided into two parts. The first part gathers participant demographic information, including participants’ age, gender, and residency level. The second part consists of 11 sections that focus on basic obstetric ultrasound training aspects and performance. These include basic obstetric ultrasound (OBUS) training status and resource material for OBUS (two items), the knowledge of OBUS (two items), confidence level (one item), barriers (eight items), satisfaction level of residents (one item), suggestions to improve (one item), proficiency of OBUS (one item), number of scans per week (one item), pregnancy-associated complications detection (four items), ultrasound practice (five items) on antenatal care and resources to improve OBUS at PHCC (three items).

Our study included four open-ended questions in the form of continuous variables, namely, the age of the participant, suggestions to improve OBUS, and the number of scans required to achieve proficiency. For all the open-ended questions, we kept the option of filling in the blanks, so participants wrote their answers. Regarding the open-ended question of suggestions to improve OBUS, some participants gave more than one suggestion. Hence, the total number of responses to this question was 64. As it is an open-ended question, the number of responses is different and dispersed. Then, all the responses were analyzed manually based on certain themes, such as more ultrasound training for residents, trained specialist teachers, availability of ultrasound, booking the patient in advance, patient accepting male physicians to examine, more machines of ultrasound, practice by ultrasound supervisor, mandatory antenatal clinic in FM rotation, more time for training, advanced ultrasound device, and OBUS training in radiology rotation. Some residents did not say anything about the improvement in OBUS.

Out of 28 closed-ended questions, 24 questions are two options (yes/no option). For closed-ended questions, we kept two options (yes/no option). Gender questions are male/female options, and resident-level questions are R1/R2/R3 options. One confidence question had three options, such as not confident, neutral, and confident. The overall satisfaction level with performing OBUS for ANC patients is based on a scale of 1 to 10. Two questions are multiple-choice.

Sample size and sampling

Qassim family medicine residents at selected primary healthcare centers in Buraidah City and Unaizah City, Saudi Arabia, were chosen to participate in the survey. Based on the Qassim Family Medicine Program, there are 62 family residents, including all 3 years. Hence, our sample is a universal number of 62 residents. The survey questionnaire was distributed via a Google link via e-mail; after obtaining informed consent on the first page of the link, the actual questionnaire started. The study did not target a particular age range or particular gender. However, the participants had to be family medicine residents working at different PHCCs in Qassim province.

Inclusion criteria and exclusion criteria

Primary health care physicians belong to Qassim province FM residents and are of both genders were included. Other physicians, such as specialists and consultants working at PHCC, will be excluded. Non-cooperative participants and current R1 residents (2024-25 batch) were excluded as they were new to the program.

Pilot study

A pilot study was conducted in the field among 10 physicians to determine technical feasibility and ensure a good presentation and order of the questions. The pilot study sample was not included in the main study sample. After the pilot study, we did not change the questionnaire variables and process of the research.

Ethical considerations

Ethical committee approval was obtained from the Reginal Ethics Committee, Qassim, with approval number 607-45-15485 dated 27.05.2024. After the ethical committee's approval, data collection started. Every participant gave informed consent while filling out the Google link, and no harm was involved in this study. Confidentiality of the individual information was maintained. Individual data were not shared with any public or private agency.

Statistical analysis

Data was transferred from Microsoft Excel to SPSS v. 21.0 (IBM Corp., Armonk, NY, US). Then, the data were cleaned, coded, and analyzed with SPSS. Means and standard deviations were calculated for the continuous variables in the study. Three years of residents were categorized into two categories by sub-group analysis: junior level (R1 and R2) and senior level (R3 residents). For all the categorical variables, the chi-square test was applied. The level of statistical significance was taken as the probability (P) value is less than or equal to 0.05.

## Results

Fifty Qassim family medicine residents participated in the current study. We distributed our questionnaire through a Google link to all 62 family medicine residents who work at the different PHCCs and hospitals for their clinical rotations in Qassim province. The response rate in the study population was 80.64% (50/62). We made maximum efforts to complete our study and achieve the best response rate. The mean overall satisfaction of the FM residents about basic OBUS was about 4.62 ± 2.01 (range is 7). Similarly, the mean average obstetric ultrasound scans done per week question among the residents; the mean and SD were 6.35 ± 8.53 (range of scans 30). The residents responded to the question of the number of scans to be done to get the proficiency OBUS; the mean and SD were 19.85 ± 24.88 (range is 100). Tables 1-8 show the results of our study.

**Table 1:** Demographic characteristics of the FM residents working at the PHCCs of Qassim The table shows that the FM residents' mean age and standard deviation (SD) were 28.62 ± 2.21. The maximum number of participants was from R3 residents, which accounts for 44% (22/50), and 58% were from the male gender. PHCC: primary health care center

Demographic variables	Number of participants	Percentage
Age in years ± SD	28.62 ± 2.21 years	
Age category (n=50)		
25-30 years	42	84.0
31-35 years	5	10.0
Not mentioned age	3	6.0
Gender (n=50)		
Male	29	58.0
Female	21	42.0
Level of resident (n=50)		
R1	15	30.0
R2	13	26.0
R3	22	44.0

**Table 2:** Training aspects and confidence status of the FM residents about obstetric ultrasound in the study group The table states that about 56% (n=28) of participants received training for basic obstetric ultrasound, and 78% (n=39) revealed that they did not receive resource material for OBUS. Approximately 68% (n=34) were revealed as neutral about confidence about the psychomotor skills of OBUS, and only 6% (n=3) were mentioned as confident about OBUS psychomotor skills. FM: family medicine; OBUS: obstetric ultrasound

Training aspects of residents	No (%)	Yes (%)	Not answered (%)
Have you received any training for Obstetric Ultrasound (OBUS)?	21 (42%)	28 (56%)	01 (2%)
Have you received any resource material for OBUS?	39 (78.0%)	11 (22.0%)	0 (0%)
Confident in psychomotor skills of OBUS (n=49)	Not Confident (%)	Neutral (%)	Confident (%)
Participants response	12 (24.0%)	34 (68.0%)	3 (6.0%)

**Table 3:** Opinions of residents about barriers while performing OBUS at PHCC The table states that 93.8% (n=46) mentioned a lack of training, almost 73% (n=35) revealed a lack of trained teachers, and patient overload (74.5%). OBUS: obstetric ultrasound; PHCC: primary health care center

Barriers	No (%)	Yes (%)
Lack of equipment (n=47)	19 (40%)	28 (60.0%)
Lack of training (n=49)	3 (6.1%)	46 (93.8%)
Lack of trained teachers (n=48)	13 (27.0%)	35 (73.0%)
Not enough time from trainers (n=49)	11 (22.4%)	38 (77.6%)
Lack of time from Residents (n=47)	28 (59.5%)	19 (40.5%)
Patient overload (n=47)	12 (25.5%)	35 (74.5%)
Ultrasound machine malfunction (n=47)	26 (55.3%)	21 (44.7%)
Ultrasound maintenance problem (n=47)	29 (61.7%)	18 (38.3%)

**Table 4:** Suggestions of residents to improve basic OBUS in the study population (n=64) The table highlights that about 26.5% (n=17) of FM residents opined that more ultrasound training for residents is needed, and 14% (n=9) suggested that trained specialist teachers are required. OBUS: obstetric ultrasound

Suggestions of Residents	Frequency (%)
More ultrasound training for residents	17 (26.5%)
Trained specialist teachers	09 (14%)
Availability of ultrasound equipment	03 (4.6%)
Booking the patient in advance	03 (4.6%)
Patients accept male physicians to examine	04 (6.25%)
More machines of ultrasound at PHC	04 (6.25%)
Practice by ultrasound supervisor	03 (4.6%)
Mandatory antenatal clinic in FM rotation	03 (4.6%)
More time for training	02 (3.1%)
Advanced ultrasound device	04 (6.25%)
OBUS training in radiology rotation (including in maternity hospital)	02 (3.1%)
Not applicable suggestion	10 (15.6%)

**Table 5:** Diagnosis status of certain pregnancy-associated complications and common antenatal practices among the study population The table shows that about 51% (n=25) of residents said they did not identify an anencephalic pregnancy, followed by placenta previa, which was about 32% (n=16). Regarding antenatal care ultrasound practice, approximately 58% (n=29) of residents find difficulty in identifying the amniotic fluid index, followed by placental location 34% (n=17).

Pregnancy complications identification	No (%)	Yes (%)
Molar pregnancy (n=50)	13 (26.0)	37 (74.0)
Anencephalic pregnancy (n=49)	25 (51.0)	24 (49.0)
Placenta previa (n=50)	16 (32.0)	34 (68.0)
Ectopic pregnancy (n=50)	14 (28.0)	36 (72.0)
Can you identify the common antenatal care ultrasound practice		
Fetal position (n=49)	11 (22.0)	38 (76.0)
Amniotic fluid index (n=49)	29 (58.0)	20 (40.0)
Placental location (n=50)	17 (34.0)	33 (66.0)
Cardiac activity (n=50)	6 (12.0)	44 (88.0)
Fetal age (n=50)	15 (30.0)	35 (70.0)

**Table 6:** Residents' opinions about the need for any resources to improve OBUS at PHCCs The table highlights that 96% (n=48) of residents responded that materials resources would improve OBUS at PHCCs, followed by manpower resources, which are about 92% (n=46). OBUS: obstetric ultrasound; PHCC: primary healthcare center

Opinions of Residents about Resources	No (%)	Yes (%)
Resources to improve OBUS at PHCC Infrastructure	6 (12.0)	44 (88.0)
Resources to improve OBUS at PHCC Materials	2 (4.0)	48 (96.0)
Resources to improve OBUS at PHCC Manpower	4 (8.0)	46 (92.0)

**Table 7:** Sociodemographic factors in relation to residents' resource material of OBUS in the study population The table states that 89.3% (n=25) of junior-level residents did not receive materials, whereas senior-level residents did not receive materials (about 63.6%; n=14). A statistically significant association was observed between junior-level residents and resource materials received status (P<0.05). OBUS: obstetric ultrasound

Sociodemographic factors	Not received resource material	Received resource material	X^2^, P value
Age category			
25-30 years	32 (76.2%)	10 (23.8%)	X^2 ^= 0.036, P=0.849
31 – 35 years	4 (80.0%)	1 (20.0%)	
Gender			X^2 ^= 0.911, P=0.340
Male	24 (82.8%)	5 (17.2%)	
Female	15 (71.4%)	6 (28.6%)	
Resident level			
Junior level	25 (89.3%)	3 (10.7%)	X^2 ^= 4.723, P=0.030
Senior level	14 (63.6%)	8 (36.4%)	

**Table 8:** Gender and residency level in relation to confidence in psychomotor skills of OBUS in the study population The table states that 32.1% (n=9) of junior residents were not confident about the psychomotor skills of OBUS, whereas only 14.3% (n=3) of senior residents were not confident about the psychomotor skills of OBUS, but this association was not statistically significant (P>0.05). OBUS: obstetric ultrasound

Gender	Not confident	Neutral	Confident	X^2^, P value
Male	7 (25.0%)	20 (71.4%)	1 (3.6%)	X^2 ^= 0.741, P=0.691
Female	5 (23.8%)	14 (66.7%)	2 (9.5%)	
Resident level				
Junior level	9 (32.1%)	17 (60.7%)	2 (7.1%)	X^2 ^= 2.382, P= 0.304
Senior level	3 (14.3%)	17 (81.0%)	1 (4.8%)	

## Discussion

Ultrasound is the most often used diagnostic technique in obstetrics. It is considered safe, easy to use, painless, and shows immediate outcomes [14]. However, it is an operator-dependent imaging modality. The value of ultrasound examination is primarily dependent on the operator's skill, experience, and preparation. Examiner performance directly impacts patient diagnosis and treatment [15,16].

Knowing the confidence levels helps ensure that physicians are adequately trained to perform ultrasounds accurately, which is crucial for patient safety and optimal care. The current cross-sectional study was conducted with the objective of perceptions of the basic OBUS performance among the Qassim FM residents working at different PHCCs of Qassim. Good confidence levels in FM doctors' OBUS skills enhance the better outcome of early identification of maternal, fetal, and genetic abnormalities [7]. Indirectly, this facility provides the bonding between FM doctors and patients, ultimately enhancing patient satisfaction and reducing the specialist load on Obstetricians/radiologists working at secondary hospitals [17].

 In the current study, about 56% (n=28) of participants received training in basic obstetric ultrasound, while 78% (n=39) indicated that they did not receive resource materials for OBUS. Approximately 68% (n=34) reported being neutral about their confidence in the psychomotor skills related to OBUS, and only 6% (n=3) expressed confidence in their OBUS psychomotor skills. In addition, 32.1% (n=9) of junior residents expressed a lack of confidence in their psychomotor skills related to obstetric ultrasound while only 14.3% of senior residents reported a lack of confidence. The result could be explained by a cross-sectional study among members of the national societies of junior obstetricians/gynecologists in Denmark, Sweden, and Norway, which aimed to explore the association between clinical training characteristics and trainees' confidence level in performing ultrasound scans independently. They found that the duration of time spent in specialized ultrasound facilities and clinical experience were predictive of trainees' confidence in performing ultrasonography [18].

The Johnson J et al. study was conducted among family medicine residents about hands-on workshops on ultrasound and concluded that a positive correlation between confidence in performing “point of care ultrasound” (POCUS) and the implementation of a POCUS curriculum with didactic and hands-on training [19]. A prospective study at Sparrow/Michigan State University among 40 family medicine residents concluded that basic OBUS training improved their understanding and confidence, and results showed significant enhancement in knowledge and confidence post-training [12]. Another study in the United States sought to evaluate medical students' confidence levels and competencies in identifying abdominal structures by ultrasound (US) after participating in integrated US sessions. The results demonstrated a significant enhancement in both the ability and confidence of students in performing US following hands-on training sessions [20].

Approximately 93.8% (n=46) of participants identified a lack of training as a barrier to performing OBUS at primary health care centers while nearly 73% (n=35) cited a lack of trained instructors. Additionally, others mentioned patient overload as a contributing factor in our study. The result is consistent with multiple studies, and the top barrier mentioned is a lack of training [21,22].

Hall JW et al. investigated ultrasound training in family medicine residencies. They found an increase in residencies using point-of-care ultrasonography (POCUS), barriers included a lack of trained faculty, limited ultrasound equipment, and discomfort in interpreting images without radiologist review [23].

One study was conducted among healthcare providers who work with an ultrasound machine to assess perceived barriers to ultrasound. They concluded that lack of training is a primary barrier to regular use of ultrasound [12]. Another study carried out in five states in the United States (Washington, Wyoming, Alaska, Montana, and Idaho) aimed to define perceived barriers to using emergency POCUS. Emergency physicians identify a lack of training as a primary barrier to the regular use of point-of-care ultrasound in their practice [24].

Another study done in 2024 by Resnyk et al. mentioned that common obstacles to performing ultrasound consist of a lack of formal training, limited access to ultrasound machines, issues with quality assurance, insufficient time to conduct exams, and challenges in incorporating findings into documentation and decision-making. Additionally, low confidence may be an underrepresented barrier [25].

To improve OBUS, 26.5% of the participants highlighted the importance of more Ultrasound training for residents, as a key factor for enhancing their OBUS skills. Our findings were aligned with a study that took place during a training program for obstetric and gynecologic ultrasound in Muscat, Oman, which revealed that applying the International Society of Ultrasound in Obstetrics and Gynecology basic training and teaching course enhances the theoretical knowledge and practical skills of the health care worker [26].

The findings show that 96% (n=48) of residents highlight that materials resources would improve OBUS at PHCC, followed by manpower resources, which are about 92% (n=46). The result is supported by a study published in 2020 focused on developing learning resources such as a guidebook and instructional videos on basic obstetric ultrasound examinations and their influence in improving undergraduate medical students’ knowledge of basic obstetric examination skills. The results showed significant improvements in students' knowledge after introducing these materials. Implementing teaching media, including the basic obstetric ultrasound module and videos, boosted students' knowledge in this area [27].

Some of the limitations observed in our study are the small sample and data collected from the online Google form; a reasonable response rate was observed. The study's major strength is that more than 80% of resident samples were collected and OBUS is one of SCFHS's recommendations for the FM program. Also, being an FM specialty, there has been an enormous benefit to the community over the years of FM practice and facility provision of ultrasound at the PHCCs.

## Conclusions

In conclusion, FM residents need training, access to resource materials, specialist doctor training, and periodical retraining, as ultrasound is a skill development procedure. Senior residents feel more confident about performing OBUS than junior residents, likely due to their greater practice and exposure. Some residents suggested that a radiology rotation could be conducted between King Fahad Hospital and Maternity Hospital to get more antenatal ultrasound exposure. We recommend that administrators and policymakers enhance FM residents' OBUS performance.

## Figures and Tables

**Figure 1:**
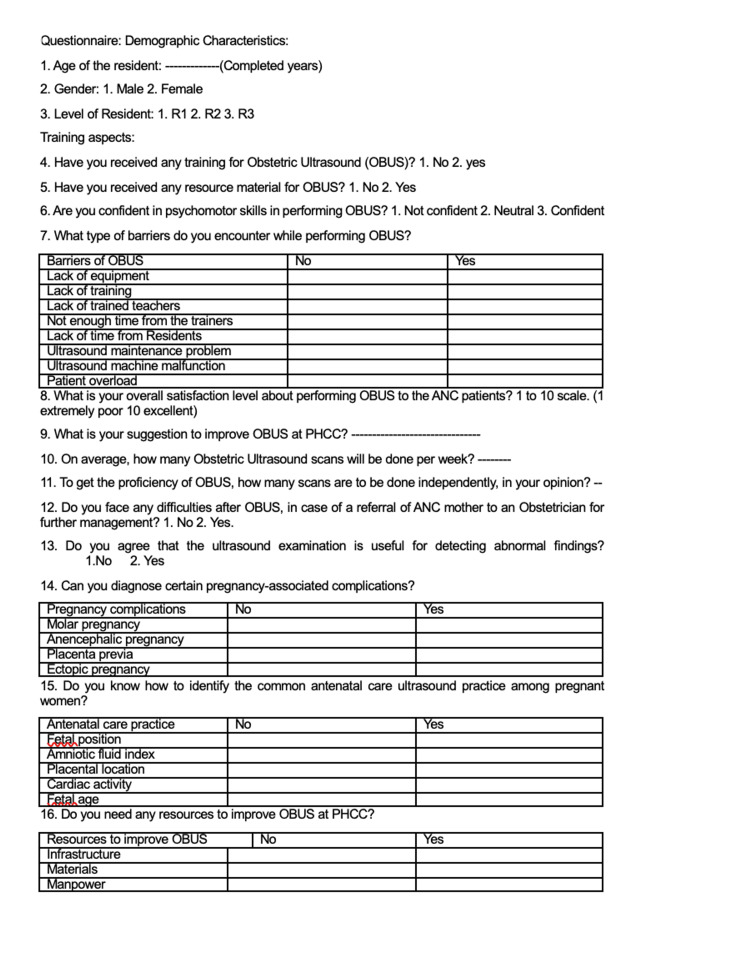
The questionnaire used in the study
